# Early Insights from Commercialization of Gene Therapies in Europe

**DOI:** 10.3390/genes8020078

**Published:** 2017-02-17

**Authors:** Nicolas Touchot, Mathias Flume

**Affiliations:** 1 groupH, Strategy Consulting, London SW15 2AW, UK; 2 Kassenärztliche Vereinigung Westfalen Lippe, Dortmund 44141, Germany; mathias.flume@gmx.de

**Keywords:** gene therapies, payers, market access, Glybera, Strimvelis, Imlygic, reimbursement, commercialization

## 1. Introduction

After years of research and development, gene therapies are now becoming a commercial reality with several products approved by European regulatory authorities. However, regulatory approval does not mean that these products are available to patients or are paid for by European health systems. In European countries, novel therapies, irrespective of the nature or mechanism of action, have to be evaluated through a formal Health Technology Assessment process, which is followed by (or includes) price negotiations. Developers of gene therapies are thus now facing the “fourth hurdle”: convincing health systems that they should invest into highly expensive products with the expectation that long-term patient benefits justify the cost.

Three gene therapies have now been approved by European regulatory authorities, Glybera, Imlygic and Strimvelis, allowing us to perform a first analysis of perceptions by payers.

In the first part of this communication, we will review the outcome of early gene therapy commercialization efforts with a focus on EU5 (Germany, France, UK, Spain and Italy) (Table 1). We will then derive initial conclusions and lessons for the developers of gene therapies.

## 2. Review of Gene Therapies Approved for Commercialization in Europe

### 2.1. Glybera

Glybera (alipogene tiparvovec), for the treatment of lipoprotein lipase deficiency, an ultra-rare disease leading to abdominal pain, pancreatitis and xanthomas, was approved in Europe in 2012. Glybera has been formally evaluated through Health Technology Assessment in Germany [1] and in France [2] but failed to achieve a recognition of benefit in either country.

In France, the HAS (Haute Authorité de Santé) Transparency Commission (The French Health Technology Assessment body) stated that:

- “A moderate effect on triglycerides and on episodes of pancreatitis has been observed but this effect was not sustained in the medium- and long-term” (in line with submitted efficacy data showing only transient efficacy);
- “The clinical relevance of the chosen primary efficacy endpoint (reduction in the triglyceride level) is debatable”;
- “Uncertainties about the short- and medium-term safety of this gene therapy, which cannot be re-administered because of its action mechanism, remain.”

As a result, the HAS concluded that the actual benefit of Glybera is insufficient to justify reimbursement by their national health insurance. The product is therefore not commercialized in France.

In Germany, Glybera was initially positioned as a community product as it does not require hospital admission for administration. It was thus formally evaluated through AMNOG (the German Health Technology Assessment process) and was granted “unquantifiable additional benefit”. Orphan drugs receive an additional benefit by legal definition in Germany. G-BA (German Federal Joint Committee) concluded that clinical data did not support an additional benefit, and thus the “unquantifiable” category was chosen. The positioning was then changed to a hospital-only product, allowing direct price negotiations between hospitals and payers. While these negotiations are highly resource-intensive and time-consuming, national price negotiations and coverage decisions are avoided. Direct hospital/payer negotiations have been used for the single patient treated so far at Charité in Berlin in September 2015. The price was about €900,000 following an agreement with DAK (Deutschen Angestellten-Krankenkasse), a large German health insurance provider. So far, other German health insurances have not followed the DAK example, which is often considered a “marketing coup” rather than a true endorsement of Glybera. Other German payers have described the process “urban warfare” and have expressed concerns about the “publicity” given to such cases as it could drive patients in need of gene therapy to change Sick Funds (German Health Insurances), flooding to those with a history of coverage. The “pull” from physicians and patients for Glybera was also very limited.

Glybera has not been evaluated and is unlikely to ever be commercialized in the UK or in Italy. No patient has been “commercially” treated (with reimbursement/payment for the product) in other European countries. So now, more than four years after European regulatory approval, the first commercial gene therapy has been used in only one case. Plans for US commercialization have been abandoned and Chiesi, the current market authorization holder, has announced that it will identify potential patients in Europe but has not developed an approach allowing additional commercial use.

### 2.2. Imlygic

Imlygic (Talimogene laherparepvec) is a modified form of the herpes simplex virus type 1 for the local treatment of unresectable cutaneous, subcutaneous and nodal lesions in patients with melanoma recurrent after initial surgery. It was approved by both the European Medical Agency and the US Food and Drug Administration in October 2015.

The only European country in which an evaluation of Imlygic has been completed so far is the UK [3]. In an initial guidance, NICE (National Institute of Clinical Excellence) concluded that Imlygic was not cost-effective and pointed out the lack of supporting evidence and the uncertainty of overall survival compared to other drugs used to treat advanced melanoma. The acquisition cost of Imlygic was £1,670 per 10^8^ PFUs (Plaque Forming Units).

However, the company provided additional evidence and agreed to a patient access scheme with the Department of Health. This scheme provides a simple discount (confidential) to the list price of Imlygic, with the discount applied at the point of purchase or invoice. NICE also significantly narrowed the indication for coverage, restricting it to patients for whom treatment with systemically administered immunotherapies is not suitable.

Imlygic is in the process of being evaluated in other countries. In Germany, IQWiG (the German health technology assessment body) concluded that the manufacturer dossier contained no data suitable for assessment [4]. For the assessment of the added benefit, the Federal Joint Committee (G-BA) defined three treatment situations and specified a different appropriate comparator therapy for each of them. These appropriate comparator therapies depend on the pretreatment and on whether or not the gene for the BRAF enzyme has mutated in the tumor cells. For all three situations, Imlygic was compared by the manufacturer against the granulocyte-macrophage colony-stimulating factor (GM-CSF), which did not concur with the comparator specified by the G-BA for any of the research questions, and is also not approved for the treatment of melanoma. Therefore, it is likely that the final G-BA verdict will also be “no quantifiable additional benefit” with the consequence of a reimbursement price on the comparator level.

While Imlygic may achieve coverage in several European countries, it will clearly face challenges and this coverage may either be limited, or may require a price discount or both. As the HTA (Health technology Assessment) frameworks usually value outcomes and not the mode of action, the pricing potential is likely to remain limited unless additional data are produced to better support incremental efficacy to the current standard of care (as defined by payers).

### 2.3. Strimvelis

Strimvelis was developed by GlaxoSmithKline in collaboration with a charitable organization (Italian’s Fondazione Telethon) and an academic center (Ospedale San Raffaele in Milan). It is the first ex vivo stem cell gene therapy and is indicated to treat patients with ADA-SCID (severe combined immunodeficiency due to adenosine deaminase deficiency). Children born with ADA-SCID have profound lymphopenia and very low immunoglobulin levels, causing severe and recurrent opportunistic infections. It was approved in Europe in May 2016.

Strimvelis requires specific cell processing capabilities and must be infused back into the patient within a short timeframe. So far, only one site has been approved for manufacturing in Italy (Molmed), close to Ospedale San Raffaele, and patients from other European countries are expected to travel to Italy for treatment. As a result, so far the product has only been evaluated in Italy.

AIFA (Agenzia Italiana del Farmaco), the Italian medicines agency, has agreed to reimburse the treatment at a price of €594,000. This is significantly less than the price of long-term enzyme replacement therapy and is supplemented by a limited risk-share scheme as is common with specialty medicines in Italy with payback in case of treatment failure.

Strimvelis has yet to be evaluated in other European countries and the jury is still out as to whether the Italian price can be maintained. However, the very short time between approval and reimbursement agreement in Italy illustrates the quality of the clinical development and the strength of the data based on long-term survival. All treated patients whose data supported the regulatory submission were alive after a median follow-up of seven years in an indication where the expected survival is usually below one year. This confirms that with a very precise target in a monogenic disorder, along with strong data and a realistic price expectation, it is possible to get favorable coverage for a gene therapy.

While Strimvelis revenues will remain limited due to the small size of the patient population (about 15 patients per year in Europe), it represents a successful case study to follow by other developers of gene therapies.

## 3. Conclusions

The examples of Glybera and Imlygic clearly illustrate that, at least in Europe, gene therapies will be evaluated exactly as other therapies are. European health technology assessment bodies and public healthcare systems will apply the same criteria and the same scrutiny to the quality of data compared to small molecules or biologics, regardless of the very limited number of eligible patients often found in indications targeted by gene therapies. In contrast, de-risking the value to payers appears to be a fundamental feature of the success of Strimvelis. Key to this was agreeing on a simple measurable definition of “failure”.

While gene therapies are recognized as innovative and cutting-edge medicines, European health systems mainly focus on clinically significant and relevant patient outcomes compared to existing standard of care therapies, rather than on the mechanism of action used to generate these benefits. In order to meet current assessment paradigms, these data/outcomes would need to be produced for gene therapies as well, and failure to provide respective data is likely to lead either to limited or reduced levels of reimbursement, if not to rejection of reimbursement.

European health systems are also unlikely to accept the long-term benefits of gene therapies without clear proof of sustainability. Extrapolations and modeling are rejected in most cases for any drug therapies and will most likely be rejected for gene therapies as well. The exception remains the UK where NICE has issued a clear framework on extrapolation methodology [5].

The example of Strimvelis, however, shows that when long-term and robust data are generated instead of rushing products to market with fewer and less robust data and limited follow-up, rapid reimbursement at reasonable price levels can be achieved and risk-sharing strategies can be included in the reimbursement strategy.

The quality of clinical development remains paramount for gene therapies, as it is for any class of drugs. Early dialogue with payers should be routinely used to optimize and align the clinical development program for EMA/FDA approval with outcomes of relevance to payers. Gene therapies should then not be rushed to market but companies should gather the required data with the appropriate duration of follow-up to allow proper evaluation by payers. In addition, it is key to think about potential reimbursement and pricing strategies, including risk sharing, as soon as the early clinical development phase.

## Figures and Tables

**Table 1:** Commercialization status of gene therapies in key European countries.

Commercialization Status	Product	Glybera	Imlygic	Strimvelis
	EMA Approval	July 2012	October 2015	May 2016
Country (as of Nov 2016)	France	−	−	−
	Germany	+ (1 case)	+	−
	UK	−	−	−
	Italy	−	−	+ *
	Spain	−	−	−

* So far, Strimvelis can only be administered in one center in Italy for technical reasons.
